# Information Transfer Among the Components in Multi-Dimensional Complex Dynamical Systems

**DOI:** 10.3390/e20100774

**Published:** 2018-10-09

**Authors:** Yimin Yin, Xiaojun Duan

**Affiliations:** College of Liberal Arts and Sciences, National University of Defense Technology, Changsha 410072, China

**Keywords:** Information transfer, continuous flow, discrete mapping, Lorenz system, Chua’s system

## Abstract

In this paper, a rigorous formalism of information transfer within a multi-dimensional deterministic dynamic system is established for both continuous flows and discrete mappings. The underlying mechanism is derived from entropy change and transfer during the evolutions of multiple components. While this work is mainly focused on three-dimensional systems, the analysis of information transfer among state variables can be generalized to high-dimensional systems. Explicit formulas are given and verified in the classical Lorenz and Chua’s systems. The uncertainty of information transfer is quantified for all variables, with which a dynamic sensitivity analysis could be performed statistically as an additional benefit. The generalized formalisms can be applied to study dynamical behaviors as well as asymptotic dynamics of the system. The simulation results can help to reveal some underlying information for understanding the system better, which can be used for prediction and control in many diverse fields.

## 1. Introduction

Uncertainty quantification in complex dynamical systems is an important topic in prediction models. By integrating information-theoretic methods to investigate potential physics and measure indices, the uncertainty can be quantified better in ensemble practical predictions of complex dynamical systems. For instance, one of the important motivations is the couplings among variables of dynamical systems generating information at a nonzero rate [1], which produces information exchange [2]. Entropy can be used to quantitatively describe production, gathering, exchange and transfer of information [3]. Information transfer analysis can be used to detect asymmetry in the interactions of subsystems [1,4]. The emergent phenomena cannot be simply derived or solely predicted from the knowledge of the structure or from the interactions among individual elements in complex systems [5]. The dynamics of information transportation plays a critical role in complex systems, resulting in the system prediction [6,7], controls of a system [8,9] and causal analysis [10,11]. It emphasizes further understanding and investigating information transportation in complex dynamical systems. It has been applied to quantify nonlinear interactions based on the information transfer by several underlying efficient estimation strategies in complex dynamical systems [12,13,14]. Simple examples are used to illustrate various complex phenomena. The formalisms about information transfer are mostly based on two time series [1,15,16,17].

Recently, a new approach on information flow between the components of two-dimensional (2D) systems was adapted by Liang and Kleeman [6], which can be used to deal with the change of the uncertainty of one component given by the other component. This idea is based on specific interactions between two components in complex dynamical systems. For a system with dynamics given, a measure of information transfer can be rigorously formulated (referred as LK2005 formalism henceforth in [6]). In the forms of continuous flows and discrete mappings, the information flow has been analyzed using the Liouville equations [18] and the Frobenius–Perron operators [18]. These two equations are the evolution equations of the joint probability distributions, respectively. The present formalism is consistent with the transfer entropy of Schreiber [1] in both transfer asymmetry and quantification. A variety of generalizations and applications of the work in Reference [4] are developed in [19,20,21,22,23,24,25]. Majda and Harlim [26] applied the strategy to study subspaces of complex dynamical systems. For 2D systems, Liang and Kleeman discovered a concise law on the entropy evolution of deterministic autonomous systems and obtained the time rate of information flow from one component to the other [6]. Until now, the 2D formalism has been extended to some dynamical systems in different forms and scales with successful applications between two variables [23,25]. In the light of these applications, by thoroughly describing the statistical behavior of a system, this rigorous LK2005 formalism has yielded remarkable results [3].

However, the uncertainty of many real-world systems needs to be quantified among the variables for revealing the nonlinear relationships, so as to better understand the intrinsic mechanism and predict the forthcoming states of the systems [27]. Besides, many physical systems are affected by the interactions between multiple components in diverse fields [28]. For example, sensitivity analysis of an aircraft system with respect to design variables, parameters and uncertainty factors can be used to estimate the effects on the objective function or constraint function. The uncertainty analysis and sensitivity analysis (UASA) process is one of the key steps for determining the optimal search direction and guiding the design and decision-making, which aims at predicting complex computer models by quantifying the sensitivity information of the coupling variables. It can be offered to quick guide of determining design parameters which lead to high performance aircraft designs. Some preceding tools [29,30] related to sensitivity analysis are applicable for low-dimensional static problems and an urgent problem of high dimensionality arises when outputting variables of numerical models with spatially and temporally need to be solved [31]. The rigorous formalism of information flow has the potential to revolutionize the ability to analyze and measure uncertainty and sensitivity information in dynamical systems.

Hence, considering realistic applications, we generalize the LK2005 formalism to several variables of multi-dimensional dynamical systems in this paper. More precisely, we extend the results in [6,25] to the information flow between groups of components, rather than individual components. We aim to demonstrate that the formalism is feasible among several variables in arbitrary multi-dimensional dynamical systems when dynamics is fully known. In addition, the generalized formalisms can be reduced to two-dimensional formalisms as a special case. We also highlight the relationship between the LK2005 formalism and our generalized formalisms. Two applications are proposed with the classical Lorenz system and Chua’s system as validations of our formalisms. Compared with the LK2005 formalism and the transfer mutual information method [32], the generalized formalisms are beneficial for revealing more information among variables. It can better explore the complexity of evolution and intrinsic regularity of multi-dimensional dynamical systems. Meanwhile, it can provide a simple and versatile method to analyze sensitivity in dynamical models. These generalized formulas enable one to understand the relationship between information transfer and the behavior of a system. It can be used to perform sensitivity analysis as a measure in multi-dimensional complex dynamical systems. Therefore, the generalized formalisms have much wider applications and are significant to investigate real-world problems.

The structure of this paper is as follows: Section 2 recalls a systematic introduction of the theories and the formalisms about information flow in 2D systems; In Section 3, the formalisms are generalized to adapt to multi-dimensional complex dynamical system components based on the LK2005 formalism. Details on the derivations of the formalisms and the related properties are demonstrated; Section 4 gives a description about the formalisms with multi-dimensional applications; the summary of this paper is given in Section 5.

## 2. Two-Dimensional Formalism of Information Transfer (the LK2005 Formalism [6])

### 2.1. Continuous Flows

For 2D continuous and deterministic autonomous systems with fully known dynamics,

(1) $$\frac{dx}{dt}=F\left(x\right),$$

where $F=(F_{1},F_{2})$ with $F_{i}=F_{i}\left(x_{1},x_{2}\right)$ for any i=1,2 is known as the flow vector and $x=\left(x_{1},x_{2}\right)\in \Omega =\Omega_{1}\times \Omega_{2}.$ A stochastic process $X=(X_{1},X_{2})\in \Omega$ with joint probability density $\rho (x_{1},x_{2},t)$ at time *t* is the random variables corresponding to the sample values $\left(x_{1},x_{2}\right)$. For convenience, we will use the notation ρ or $\rho (x_{1},x_{2})$ instead of the notation $\rho (x_{1},x_{2},t)$ throughout Section 2, including the same expression at multi-dimensional cases in Section 3. In addition, the integral domain is the whole sample space Ω, except where noted. The probability density ρ associated with Equation (1) satisfies the Liouville equation [18]:

(2) $$\frac{\partial \rho }{\partial t}+\frac{\partial (F_{1}\rho )}{\partial x_{1}}+\frac{\partial (F_{2}\rho )}{\partial x_{2}}=0.$$

The rate of change of joint entropy of $X_{1}$ and $X_{2},$ $H\left(t\right)\overset{def}{=}-{\;\int \int \;}_{\Omega }\rho \log\rho dx_{1}dx_{2},$ satisfies the relation [6]

(3) $$\frac{dH}{dt}=E\left(\nabla \cdot F\right),$$

where *E* means the mathematical expectation with respect to ρ and $E\left(\nabla \cdot F\right)={\;\int \int \;}_{\Omega }\rho \left(\nabla \cdot F\right)dx_{1}dx_{2}.$ That is to say, when a system evolves with time, the change of its joint entropy is totally controlled by the contraction or expansion of the phase space [6]. Later on, Liang and Kleeman showed that this property holds for deterministic systems of arbitrary dimensionality [20].

Liang and Kleeman [6] provided a very efficient heuristic argument to describe the decomposition of the various evolutionary mechanisms of information transfer in terms of the individual and joint time rates of entropy changes of $X_{1},X_{2}$ and $(X_{1},X_{2}).$ Firstly, they computed $\frac{dH_{1}}{dt}$ and $\frac{dH_{2}}{dt}$, where $H_{i}$ is the entropy of $X_{i}$ defined according to the marginal density, $\rho_{i}.$ Secondly, they employed the novel idea of frozen variables to analyze the individual time rates of entropy changes. When $X_{i}$ is fixed and $X_{j}$ evolves on its own in 2D systems, they found its temporal rate of change of entropy depends only on $E(\frac{\partial F_{j}}{\partial x_{j}}),$ denoted by $\frac{dH_{j}^{*}}{dt}.$ In the presence of interactions between $X_{i}$ and $X_{j}$, they observed that $\frac{dH_{j}}{dt}\neq E\left(\frac{\partial F_{j}}{\partial x_{j}}\right)=\frac{dH_{j}^{*}}{dt}.$ Therefore, Liang and Kleeman [6] concluded that the difference between $\frac{dH_{j}}{dt}$ and $E\left(\frac{\partial F_{j}}{\partial x_{j}}\right)$ should equal to the rate of entropy transfer from $X_{i}$ to $X_{j}.$ In the meantime, they denoted the rate of flow from $X_{i}$ to $X_{j}$ by $T_{i\to j}$ (*T* stands for ”transfer”) and defined information flow/transfer as

(4) $$T_{i\to j}=\frac{dH_{j}}{dt}-\frac{dH_{j}^{*}}{dt}=-{\;\int \int \;}_{\Omega }\rho_{i|j}\left(x_{i}|x_{j}\right)\frac{\partial (F_{j}\rho_{j})}{\partial x_{j}}dx_{i}dx_{j},$$

where $\rho_{i|j}\left(x_{i}|x_{j}\right)=\frac{\rho (x_{i},x_{j},t)}{\rho (x_{j},t)}$ and i,j=1,2 with different i,j at the same time.

### 2.2. Discrete Mappings

Similarly, Liang and Kleeman [6] also gave the formalism about a system in the discrete mapping form. Considering a 2D transformation

$$\Phi :\Omega \to \Omega ,\left(x_{1},x_{2}\right)\to \left(\Phi_{1}\left(x\right),\Phi_{2}\left(x\right)\right),$$

where $x=(x_{1},x_{2})\in \Omega$ and $\Omega :=\Omega_{1}\times \Omega_{2}.$ The evolution of the density of Φ is driven by the Frobenius–Perron operator (F−P operator) $P:L^{1}\left(\Omega \right)\to L^{1}\left(\Omega \right)$ [18]. The entropy increases as

$$\begin{array}{cc} \Delta H & =-\;\int \int \;P\rho \log P\rho dx_{1}dx_{2}+\;\int \int \;\rho \log\rho dx_{1}dx_{2}\\ & =-\;\int \int \;\rho \left(x_{1},x_{2}\right)\log\left|J^{-1}\right|dx_{1}dx_{2}, \end{array}$$

where $J^{-1}$ is the Jacobian matrix of Φ. When $\Phi_{j}$ is invertible in 2D transformations,

(5) $$\Delta H_{j}^{*}=E\log\left|J_{j}\right|.$$

The entropy of $X_{j}$ increases as

$$\Delta H_{j}=-\int_{\Omega_{j}}\left(\int_{\Omega_{i}}P\rho dx_{i}\right)\log\left(\int_{\Omega_{i}}P\rho dx_{i}\right)dx_{j}+\int_{\Omega_{j}}\rho_{j}\log\rho_{j}dx_{j},$$

where $\rho_{j}$ is the marginal density of $X_{j}.$ When $\Phi_{j}$ is noninvertible in 2D transformations,

(6) $$\begin{array}{cc} \Delta H_{j}^{*}= & \int \rho_{j}\left(x_{j}\right)\log\rho_{j}\left(x_{j}\right)dx_{j}\\ & -\;\int \int \;P_{j}\rho_{j}\left(\Phi_{j}\left(x_{i},x_{j}\right)\right)\log P_{j}\rho_{j}\left(\Phi_{j}\left(x_{i},x_{j}\right)\right)\rho \left(x_{i}|x_{j}\right)\left|J_{j}\right|dx_{i}dx_{j}, \end{array}$$

where $P_{j}$ is the F−P operator when $x_{i}$ is frozen as a parameter in $P_{j}$. The entropy transferring from $X_{i}$ to $X_{j}$ is

(7) $$\begin{array}{cc} T_{i\to j}= & -\int_{\Omega_{j}}\left(\int_{\Omega_{i}}P\rho dx_{i}\right)\log\left(\int_{\Omega_{i}}P\rho dx_{i}\right)dx_{j}\\ & +\;\int \int \;P_{j}\rho_{j}\left(\Phi_{j}\left(x_{i},x_{j}\right)\right)\log P_{j}\rho_{j}\left(\Phi_{j}\left(x_{i},x_{j}\right)\right)\rho \left(x_{i}|x_{j}\right)\left|J_{j}\right|dx_{i}dx_{j}, \end{array}$$

where i,j=1,2 with different i,j at the same time.

## 3. *n*-Dimensional Formalism of Information Transfer

### 3.1. Continuous Flows

Firstly, we consider a three-dimensional (3D) continuous autonomous system,

(8) $$\frac{dx}{dt}=F\left(x\right),$$

where $F=(F_{1},F_{2},F_{3})$ is a known flow vector. Similarly, the probability density ρ associated with Equation (8) satisfies the Liouville equation [18]:

(9) $$\frac{\partial \rho }{\partial t}+\frac{\partial (F_{1}\rho )}{\partial x_{1}}+\frac{\partial (F_{2}\rho )}{\partial x_{2}}+\frac{\partial (F_{3}\rho )}{\partial x_{3}}=0.$$

Analogous to the derivation in [6], firstly, multiplying by (1+logρ) for Equation (9), after some algebraic manipulations:

(10) $$\frac{\partial (\rho \log\rho )}{\partial t}+F\cdot \left(\nabla \cdot (\rho \log\rho )\right)+\rho \left(1+\log\rho \right)\nabla \cdot F=0.$$

Then, integrating for Equation (10),

$$\frac{dH}{dt}-{\;\int \int \int \;}_{\Omega }\nabla \cdot \left(\rho \log\rho F\right)dx_{1}dx_{2}dx_{3}-{\;\int \int \int \;}_{\Omega }\rho \nabla \cdot Fdx_{1}dx_{2}dx_{3}=0.$$

Assuming that ρ vanishes at the boundaries (the compact support assumption for ρ and the assumption is reasonable in real-world problems [6]), it is found that the time rate of the joint entropy change of $X_{1},X_{2}$ and $X_{3},$

$$H\left(t\right)\overset{def}{=}-{\;\int \int \int \;}_{\Omega }\rho \log\rho dx_{1}dx_{2}dx_{3},$$

satisfies

$$\frac{dH}{dt}-{\;\int \int \int \;}_{\Omega }\rho \left(x_{1},x_{2},x_{3}\right)\nabla \cdot Fdx_{1}dx_{2}dx_{3}=0$$

or

$$\frac{dH}{dt}=E\left(\nabla \cdot F\right),$$

where $E\left(\nabla \cdot F\right)={\;\int \int \int \;}_{\Omega }\rho \left(\nabla \cdot F\right)dx_{1}dx_{2}dx_{3}.$

As mentioned above, the time rate of change of *H* equals to the mathematical expectation of the divergence of the flow vector F. When we are interested in the entropy evolution of a component, $x_{k}$ in 3D systems, the marginal density is

$$\rho_{k}\left(x_{k},t\right)={\;\int \int \;}_{\Omega_{i}\times \Omega_{j}}\rho \left(x_{i},x_{j},x_{k},t\right)dx_{i}dx_{j}.$$

The evolution equation of $\rho_{k}$ is derived by taking the integral of Equation (9) with respect to $x_{i}$ and $x_{j}$ over the subspace $\Omega_{i}\times \Omega_{j}:$

$$\frac{\partial \rho_{k}}{\partial t}+\frac{\partial }{\partial x_{k}}{\;\int \int \;}_{\Omega_{i}\times \Omega_{j}}\rho F_{k}dx_{i}dx_{j}=0.$$

The third and fourth terms in Equation (9) have been integrated out with the compact support assumption for ρ. So the entropy for the component

$$H_{k}\left(t\right)=-\int_{\Omega_{k}}\rho_{k}\log\rho_{k}dx_{k}$$

evolves as

$$\frac{dH_{k}}{dt}={\;\int \int \int \;}_{\Omega }\left[\log\rho_{k}\frac{\partial (\rho F_{k})}{\partial x_{k}}\right]dx_{i}dx_{j}dx_{k},$$

i.e.,

(11) $$\frac{dH_{k}}{dt}=-{\;\int \int \int \;}_{\Omega }\rho \left[\frac{F_{k}}{\rho_{k}}\frac{\partial \rho_{k}}{\partial x_{k}}\right]dx_{i}dx_{j}dx_{k}.$$

The Equation (11) states how $H_{k}$ evolves with time. The evolutionary mechanism of $H_{k}$ derives from two parts: One is from the evolution itself, $\frac{dH_{k}^{*}}{dt};$ another from the transfers of $X_{i}$ and $X_{j}$ according to the coupling in the joint density distribution ρ. From Section 2, we know that when $X_{k}$ evolves on its own, then

$$E\left(\frac{\partial F_{k}}{\partial x_{k}}\right)=\frac{dH_{k}^{*}}{dt}={\;\int \int \int \;}_{\Omega }\rho \frac{\partial F_{k}}{\partial x_{k}}dx_{i}dx_{j}dx_{k}.$$

Therefore, the rate of information flow/transfer from $X_{i},X_{j}$ to $X_{k}$ is

(12) $$\begin{array}{cc} T_{i,j\to k} & =\frac{dH_{k}}{dt}-\frac{dH_{k}^{*}}{dt}={\;\int \int \int \;}_{\Omega }\rho \left(\frac{F_{k}}{\rho_{k}}\frac{\partial \rho_{k}}{\partial x_{k}}+\frac{\partial F_{k}}{\partial x_{k}}\right)dx_{i}dx_{j}dx_{k}\\ & =-{\;\int \int \int \;}_{\Omega }\frac{\rho }{\rho_{k}}\frac{\partial (F_{k}\rho_{k})}{\partial x_{k}}dx_{i}dx_{j}dx_{k}\\ & =-{\;\int \int \int \;}_{\Omega }\rho_{i,j|k}\left(x_{i},x_{j}|x_{k}\right)\frac{\partial (F_{k}\rho_{k})}{\partial x_{k}}dx_{i}dx_{j}dx_{k}, \end{array}$$

where $\rho_{i,j|k}\left(x_{i},x_{j}|x_{k}\right)=\frac{\rho (x_{i},x_{j},x_{k},t)}{\rho (x_{k},t)}$ and i,j,k=1,2,3 with different i,j,k at the same time.

In particular, if $F_{1}=F_{1}\left(x_{1}\right)$ has no dependence on $x_{2},$ then $T_{2\to 1}=0$. There is no information transfer from random variable component $X_{2}$ to $X_{1}.$ This holds true with the transfers defined in LK2005 formalism. Obviously, in system (8), when $F_{1}$ has no dependence on $x_{2},x_{3},$ there should be no information transfer from $X_{2},X_{3}$ to $X_{1},$ but there is possibility that the transfers in other directions may be nonzero when $F_{2}$ depends on $x_{1},x_{3}$ or $F_{3}$ depends on $x_{1},x_{2}.$ This is consistent with the information transfer defined in Equation (12). As a matter of fact, an important property of the transfer is given below.

**Theorem** **1.** *If $F_{k}$ is independent of $x_{i},x_{j}$ in system (8) with different i,j,k, then $T_{i,j\to k}=0.$*

**Proof** **of** **Theorem** **1** According to the formalism of information transfer for system (8), with the notation of $F_{k}=F_{k}\left(x_{k}\right),$

$$\begin{array}{cc} T_{i,j\to k} & =-{\;\int \int \int \;}_{\Omega }\rho_{i,j|k}\left(x_{i},x_{j}|x_{k}\right)\frac{\partial (F_{k}\rho_{k})}{\partial x_{k}}dx_{i}dx_{j}dx_{k}\\ & =-\int_{\Omega_{k}}\left({\;\int \int \;}_{\Omega_{i}\times \Omega_{j}}\rho_{i,j|k}\left(x_{i},x_{j}|x_{k}\right)dx_{i}dx_{j}\right)\frac{\partial (F_{k}\rho_{k})}{\partial x_{k}}dx_{k}\\ & =-\int_{\Omega_{k}}\frac{\partial (F_{k}\rho_{k})}{\partial x_{k}}dx_{k}=0. \end{array}$$

 □

It is worth noting that, while $X_{k}$ gains information from $X_{i}$ or $X_{j}$, or $X_{i}$ and $X_{j},$ $X_{i}$ or $X_{j}$ might have no dependence on $X_{k}$ in 3D systems. An important property about information transfer is its asymmetry among the components [1]. In addition, it is interesting to note that the formalism of 3D systems can be reduced to 2D cases under the condition that one variable does not depend on another variable. For example, If the evolution of $X_{k}$ is independent of $X_{i}$, then

(13) $$\begin{array}{cc} T_{i,j\to k} & =-{\;\int \int \int \;}_{\Omega }\rho_{i,j|k}\left(x_{i},x_{j}|x_{k}\right)\frac{\partial (F_{k}\rho_{k})}{\partial x_{k}}dx_{i}dx_{j}dx_{k}\\ & =-{\;\int \int \;}_{\Omega_{j}\times \Omega_{k}}\left(\int_{\Omega_{i}}\rho \left(x_{i},x_{j},x_{k}\right)dx_{i}\right)\frac{1}{\rho (x_{k})}\frac{\partial (F_{k}\rho_{k})}{\partial x_{k}}dx_{j}dx_{k}\\ & =-{\;\int \int \;}_{\Omega_{j}\times \Omega_{k}}\rho \left(x_{j}|x_{k}\right)\frac{\partial (F_{k}\rho_{k})}{\partial x_{k}}dx_{j}dx_{k}=T_{j\to k}. \end{array}$$

In particular, when $X_{k}$ is independent of $X_{i}$ and $X_{j}$,

$$\begin{array}{c} T_{i,j\to k}=T_{i\to k}=T_{j\to k}=0. \end{array}$$

According to Theorem 1, the results are apparent. Furthermore, when $X_{k}$ depends on $X_{i}$ and $X_{j}$,

$$\begin{array}{cc} T_{i,j\to k} & =-{\;\int \int \int \;}_{\Omega }\rho_{i,j|k}\left(x_{i},x_{j}|x_{k}\right)\frac{\partial (F_{k}\rho_{k})}{\partial x_{k}}dx_{i}dx_{j}dx_{k}\\ & =-{\;\int \int \int \;}_{\Omega }\rho \left(x_{j}|x_{k}\right)\cdot \rho_{i|j,k}\left(x_{i}|x_{j},x_{k}\right)\frac{\partial (F_{k}\rho_{k})}{\partial x_{k}}dx_{i}dx_{j}dx_{k}\\ & =-\int_{\Omega_{i}}\left({\;\int \int \;}_{\Omega_{j}\times \Omega_{k}}\rho_{j|k}\left(x_{j}|x_{k}\right)\frac{\partial (F_{k}\rho_{k})}{\partial x_{k}}dx_{j}dx_{k}\right)\rho_{i|j,k}\left(x_{i}|x_{j},x_{k}\right)dx_{i}\\ & =-\int_{\Omega_{i}}T_{j\to k}\cdot \rho_{i|j,k}\left(x_{i}|x_{j},x_{k}\right)dx_{i}, \end{array}$$

or

(14) $$\begin{array}{c} T_{i,j\to k}=-\int_{\Omega_{j}}T_{i\to k}\cdot \rho_{j|i,k}\left(x_{j}|x_{i},x_{k}\right)dx_{j}. \end{array}$$

From the above derivations, we can see that our formalisms are further intensified by emphasizing the inherent relation with the formalisms in 2D systems. The information flows from two variables and the high order interactions between them to another variable are quantified by formula (12). These are generalized forms of the LK2005 formalism. In Section 4, we will validate the conclusions by the applications of all formulas in the Lorenz and Chua’s systems. Moreover, when several variables are involved, the formalisms are capable to tackle information transfers of a multi-dimensional system.

Combining the Liouville equation

(15) $$\frac{\partial \rho }{\partial t}+\frac{\partial (F_{1}\rho )}{\partial x_{1}}+\frac{\partial (F_{2}\rho )}{\partial x_{2}}+\cdots +\frac{\partial (F_{n}\rho )}{\partial x_{n}}=0,$$

with Equation (3), $\frac{dH}{dt}=E\left(\nabla \cdot F\right)$ in *n*-dimensional situations, we can generalize the formalism to *n*-dimensional continuous and deterministic autonomous systems in the same way. For example, the transfer of information from components $X_{2},X_{3},\ldots ,X_{n}$ to $X_{1}$ is

$$T_{2,3,\ldots ,n\to 1}=-\int_{\Omega }\rho_{2,3,\ldots ,n}\left(x_{2},x_{3},\ldots ,x_{n}|x_{1}\right)\frac{\partial (F_{1}\rho_{1})}{\partial x_{1}}dx_{1}dx_{2}\ldots dx_{n}.$$

Hence, Theorem 1 can be generalized to multi-dimensional cases.

### 3.2. Discrete Mappings

For a 3D transformation $\Phi :\Omega \to \Omega ,\left(x_{1},x_{2},x_{3}\right)\to \left(\Phi_{1}\left(x\right),\Phi_{2}\left(x\right),\Phi_{3}\left(x\right)\right),$ the evolution of its density is driven by the Frobenius–Perron operator (F−P operator) $P:L^{1}\left(\Omega \right)\to L^{1}\left(\Omega \right)$ [18]. Similar to the 2D case, after some efficient computations, the entropy transfer from $X_{i},X_{j}$ to $X_{k}$ in three-dimensional mappings has the following form:

(16) $$\begin{array}{cc} T_{i,j\to k}= & \Delta H_{k}-\Delta H_{k}^{*}\\ = & -\int_{\Omega_{k}}\left({\;\int \int \;}_{\Omega_{i}\times \Omega_{j}}P\rho dx_{i}dx_{j}\right)\log\left({\;\int \int \;}_{\Omega_{i}\times \Omega_{j}}P\rho dx_{i}dx_{j}\right)dx_{k}\\ & +\;\int \int \int \;P_{k}\rho_{k}\left(\Phi_{k}\left(x_{i},x_{j},x_{k}\right)\right)\log P_{k}\rho_{k}\left(\Phi_{k}\left(x_{i},x_{j},x_{k}\right)\right)\rho \left(x_{i},x_{j}|x_{k}\right)\left|J_{k}\right|dx_{i}dx_{j}dx_{k}. \end{array}$$

We also give a theorem for the discrete mappings and highlight the relationship between two-dimensional formalisms and generalized formalisms. The formalisms can be extended to high-dimensional situations as well. The detailed processes are demonstrated in Appendix A.

## 4. The Application of Multi-Dimensional Formalism of Information Transfer

### 4.1. The Lorenz System

**Figure 1:**
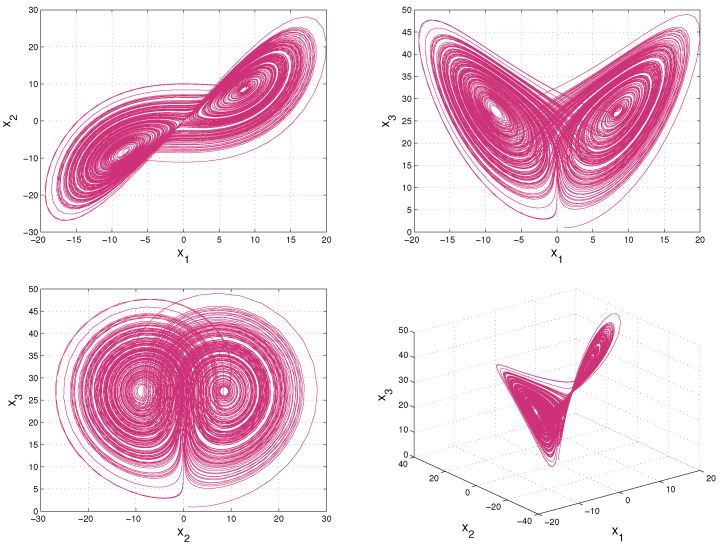
The Lorenz attractor with initial value (1,1,1).

In this section, we propose an application to study the information flows about the Lorenz system [33]:

$$\left\{\begin{array}{c} \begin{array}{c} \frac{dx_{1}}{dt}=\sigma \left(x_{2}-x_{1}\right)\\ \frac{dx_{2}}{dt}=x_{1}\left(r-x_{3}\right)-x_{2}\\ \frac{dx_{3}}{dt}=x_{1}x_{2}-bx_{3} \end{array} \end{array}\right.,$$

where σ,r and *b* are parameters, $x_{1},x_{2}$ and $x_{3}$ are the system state variables, and *t* is time. A chaotic attractor of Lorenz system with $\sigma =10,r=28,b=\frac{8}{3}$ is shown in Figure 1.

Firstly, we need to obtain the joint probability density function $\rho (x_{1},x_{2},x_{3})$ of X to calculate information flows among the variables. For a deterministic system with known dynamics, the underlying evolution of the joint density $\rho (x_{1},x_{2},x_{3})$ can be obtained by solving the Liouville equation. Taking into account of the computational load, we estimate the joint density $\rho (x_{1},x_{2},x_{3})$ via numerical simulations. The steps are summarized as follows:

- Initialize the joint density $\rho (x_{1},x_{2},x_{3})$ with a preset distribution $\rho_{0}$, then generate an ensemble through drawing samples randomly according to the initial distribution $\rho_{0}$.
- Partition the sample space Ω into “bins”.
- Obtain an ensemble prediction for the Lorenz system at every time step.
- Estimate the three-variable joint probability density function ρ via bin counting at every time step.

The Lorenz system is solved by applying a fourth order Runge–Kutta method with a time step Δt=0.01. According to Figure 1, the computation domain is restricted to Ω≡[−30,30]×[−30,30]×[0,60], which includes the attractor of the Lorenz system. We discretize the sample space into 60×60×60=21,600 bins to ensure covering the whole attractor and one draw per bin on average via making 21,600 random draws. Initially, we assume X is distributed as a Gaussian process N(u(t),Σ(t)), with a mean *u* and a covariance matrix Σ:

$$u\left(0\right)=\left[\begin{array}{c} u_{1}\\ u_{2}\\ u_{3} \end{array}\right],\Sigma \left(0\right)=\left[\begin{array}{ccc} \sigma_{1}^{2} & 0 & 0\\ 0 & \sigma_{2}^{2} & 0\\ 0 & 0 & \sigma_{3}^{2} \end{array}\right].$$

**Figure 2:**
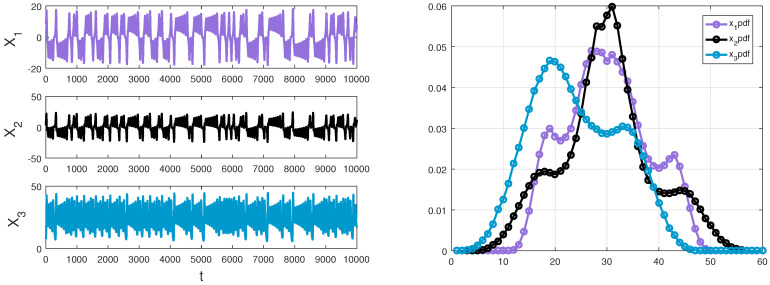
Left panel: a sample data ($X_{1},X_{2}$ and $X_{3}$) of the Lorenz system generated by a fourth order Runge–Kutta method with Δt=0.01. Right panel: an estimated marginal density of $x_{1},x_{2}$ and $x_{3}$ via counting the bins and initializing with a Gaussian distribution, respectively.

Although we have used different parameters *u* and $\sigma_{d}^{2}$ (d=1,2,3) to compute information flows for the Lorenz system, the final results are the same and the trends stay invariant. The parameters *u* and $\sigma_{d}^{2}$ can be adjusted for different experiments. Here we only show the results of one experiment with $u_{d}=4$ and $\sigma_{d}^{2}=4$. The ensemble is developed by drawing sample randomly in the light of a pre-established distribution $\rho_{0}\left(\underline{x}\right)$. We obtain an ensemble of X and estimate the three-variable joint probability density function $\rho (x_{1},x_{2},x_{3},t)$ by the way of counting the bins, at every time step. As the equations are integrated forward in the Lorenz system, ρ can be estimated as a function of time and describe the statistics of the system. A detailed discussion on probability estimation through bin counting are referred to [20,25]. The sample data with initial value (1,1,1) and an estimated marginal density of $x_{1},x_{2}$ and $x_{3}$ are displayed in Figure 2.

**Figure 3:**
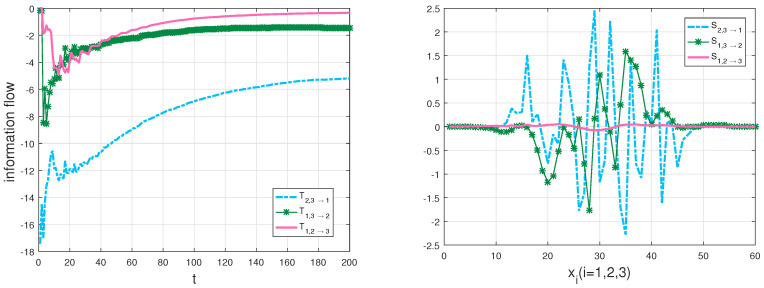
Left panel: the multivariate information flow of the Lorenz system: blue dot-dash line: $T_{2,3\to 1}$; green star line: $T_{1,3\to 2}$; red solid line: $T_{1,2\to 3}$ (in nats per unit time); Right panel: the information strength of transfer in the Lorenz system: blue dot-dash line: $S_{2,3\to 1}$; green star line: $S_{1,3\to 2}$; red solid line: $S_{1,2\to 3}$ (arbitrary unit).

Through formula (12), the information transfer within three variables can be computed. There are nine transfer series in the Lorenz system, but here we mainly focus on the couple effect from two components to another component, that is, $T_{i,j\to k},i,j,k=1,2,3$ with different i,j,k at the same time. A nonzero $T_{i,j\to k}$ means that $X_{i}$ and $X_{j}$ are causal to $X_{k}$, and the value means how much uncertainty that $X_{i}$ and $X_{j}$ bring to $X_{k}$. Among all the transfers, it is clearly shown that any two variables drive the other variable in the dynamics except the evolution of $X_{1}$ which only depends on $X_{2}.$ For the sake of revealing some underlying information in the chaotic dynamical system better, we also give information transfer among the components over space with a Gaussian distribution initialization and the averaged density over time via using the following formula: $S_{i,j\to k}=-\;\int \int \;{\bar{\rho }}_{i,j|k}\left(x_{i},x_{j}|x_{k},t\right)\frac{\partial (F_{k}{\bar{\rho }}_{k}\left(t\right))}{\partial x_{k}}dx_{i}dx_{j}$, which characterizes the strength of information transfer at different planes of $x=x_{i}$. That is to say, it demonstrates the information transfer of $x_{j}$ and $x_{k}$ to $x_{i}$ plane, whose relative values represent the magnitudes of information transfer. The calculation results are plotted in the left panel and the right one of Figure 3, respectively. According to the magnitude of parameters in the Lorenz system and the definition of rigorous 3D formalisms, the information transfer from $X_{1},X_{2}$ to $X_{3}$ is the smallest. The results are just as we expected, $\left|T_{1,2\to 3}\right|<\left|T_{1,3\to 2}\right|<\left|T_{2,3\to 1}\right|$, as shown in the left panel of Figure 3. Meanwhile, we can get much information through numerical simulations. For example, the information transfer from $X_{2}$ and $X_{3}$ on $X_{1}$ is larger than that of $X_{1}$ and $X_{3}$ on $X_{2}$ in the Lorenz system, which is helpful for us to better analyze the system and the fields of interest. Only the absolute value of *T* measures the information transfer among the variables [23]. As the ensemble evolution is carried forth, any two variables aim to reduce the uncertainty of the other variable [24]; in other words, any two variables tend to stabilize the other variable. All information flows go to constants, which means that the system tends to be stable simultaneously. Comparing the left panel with the right one in Figure 3, we can find that not only the information flow from $X_{2}$ and $X_{3}$ to $X_{1}$ is the largest at different times, but also the total information transfer is the largest at $x_{1}$ plane, and the strength of information transfer obeys a distribution in each direction of *x*. Repeated experiments are found to be in line with the results no matter whatever the initialization is given.

**Figure 4:**
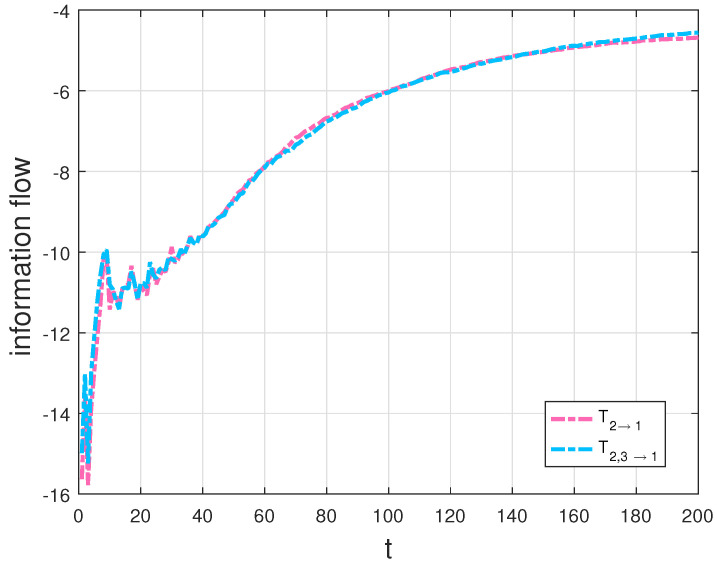
$T_{2\to 1}$ and $T_{2,3\to 1}$ in the Lorenz system (in nats per unit time).

**Figure 5:**
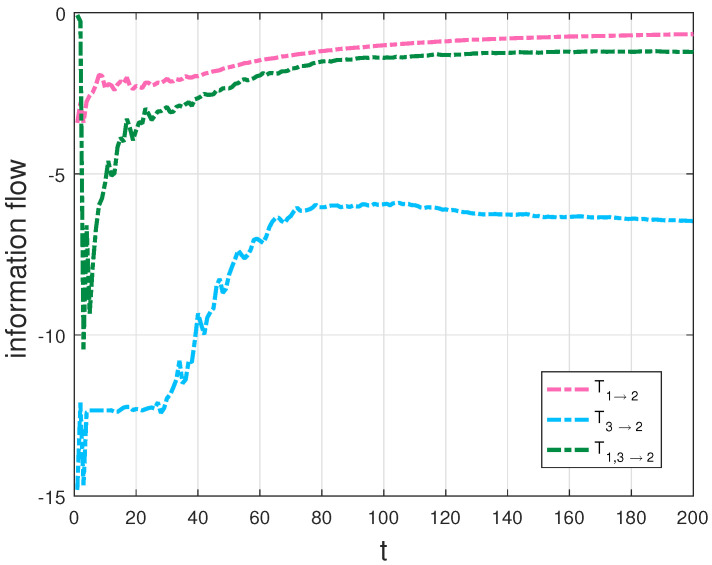
$T_{1\to 2}$, $T_{3\to 2}$ and $T_{1,3\to 2}$ in the Lorenz system (in nats per unit time).

In particular, we compute the transfer, $T_{2\to 1}$, then compare $T_{2\to 1}$ with the transfer, $T_{2,3\to 1}$ in Figure 4, as well as plot the transfers $T_{1\to 2}$, $T_{3\to 2}$ and $T_{1,3\to 2}$ in Figure 5.

Since the evolution of $X_{1}$ is independent of $X_{3}$ and the evolution of $X_{2}$ depends on $X_{1}$ and $X_{3}$ in the Lorenz system, the transfer $T_{2\to 1}$ should be equal to $T_{2,3\to 1}$ and neither the transfer $T_{1\to 2}$ nor $T_{3\to 2}$ should not be equal to $T_{1,3\to 2}$ according to the derivations in Section 3.1. As expected, there is almost no difference between the two flows in Figure 4. The interpretation of the results is that $X_{3}$ is not causal to $X_{1}$ in the Lorenz system. The result agrees well with theoretical analysis, which also validates our formalisms. But the graphs $T_{1\to 2}$ and $T_{3\to 2}$ are quite different from the graph $T_{1,3\to 2}$ in Figure 5, as that both $X_{1}$ and $X_{3}$ are causal to $X_{2}$ in the Lorenz system. From Figure 4 and Figure 5, we can find that the information flow $T_{2\to 1}$ is different from $T_{1\to 2}$, as a property of asymmetry of the information transfer. There exists hidden sensitivity information in information transfer processes of high-dimensional dynamical systems: whether or not one variable brings more uncertainty to another variable. Comparing the magnitudes of three flows in Figure 5, we can say that $X_{3}$ is more sensitive to $X_{2}$ than $X_{1}$ to $X_{2}$ from the sensitivity analysis point of view. All the above differences are exactly the embodiment of the differences between the information flows in multi-dimensional dynamical systems and the LK2005 formalism. The proposed formalisms can be used to measure information transfers among the variables in dynamical systems and the numerical results can show how the measurement behavior with time, compared with the qualification of information transfer between two variables [4] and the transfer mutual information method [32]. For example, it can be quantified the influence that $x_{3}$ on the relationship between $x_{1}$ and $x_{2}$ using the transfer mutual information method in the Lorenz system. With our generalized formalisms, we can give quantitatively the influence from $x_{3}$ to the relationship between $x_{1}$ and $x_{2}$ as a dynamical process and other relationships (such as the asymmetrical influence between two variables) among the variables for analyzing the system better. To test the influence of error propagation on the measurement of information transfers, we use a different natural interval extension to compute information transfers according to the striking method [34]. In other words, we compute information transfers using formula (12) in the Lorenz system with the rewritten second equation, that is, $rx_{1}-x_{1}x_{3}$ is used to replace $x_{1}\left(r-x_{3}\right)$. For the Lorenz system, the results show that, the algorithm performs well (the relative error < 2%). All simulations are performed on a 64-bit Matlab R2016a environment. The physical consistency of the proposed approach in this paper can be explained as that a direction of the phase space is frozen in order to extract information transfers from the other two directions [3]. In addition, nonlinearity may lead a deterministic system to chaos, which causes the “spikes” in the right panel of Figure 3 and corresponds to intermittent switching in the chaotic dynamics. As the remarkable theory stated in [35], it indicates when the dynamics are about to switch lobes of the attractor in the Lorenz system.

Since Liouville equations and Frobenius–Perron analysis describe an ensemble of trajectories, we can use the generated formalisms of information flow as a sensitivity analysis index to perform dynamic sensitivity information analysis instead of the preceding widely used methods such as repeated calculation of principal component coefficients [36,37], construction of functional metamodels [31,38], calculation of moving average of the sensitivity index [39] and direct perturbation analysis of a dynamical system [40]. Using information flow to identify sensitive variables is directly based on the statistical perspective, which can improve numerical accuracy and efficiency while reduce the calculation load, compared with conventional dynamic sensitivity analysis methods. We cannot only quantify how much the uncertainty among variables of a system, but also understand how they influence system behavior, so it may be measured and used for prediction and control in realistic applications.

**Figure 6:**
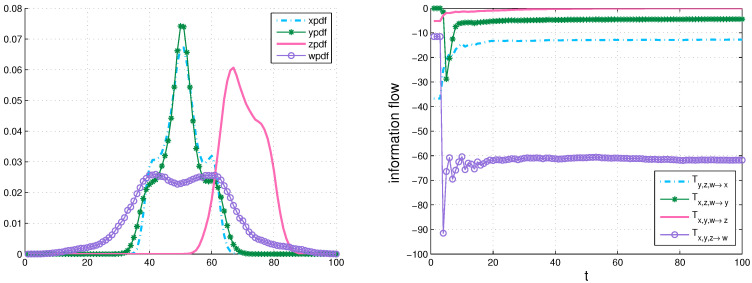
Left panel: an estimated marginal density of x,y,z and *w* via counting the bins and initializing with a Gaussian distribution, respectively; Right panel: the multivariate information flow over time of a 4D dynamical system.

Furthermore, we use Equation (15) to compute information transfers, $T_{yzw\to x}$, $T_{xzw\to y}$, $T_{xyw\to z}$, and $T_{xyz\to w}$ with the same strategy in the four-dimensional (4D) dynamical system:

$$\left\{\begin{array}{c} \begin{array}{c} \frac{dx}{dt}=12\left(y-x\right)\\ \frac{dy}{dt}=23x-xz-y+w\\ \frac{dz}{dt}=xy-2.1z\\ \frac{dw}{dt}=-6y-0.2w \end{array} \end{array}\right.,$$

whose results are shown in Figure 6.

The generalized formalisms are useful to deal with universal problems, which is not difficult to be applied to higher-dimensional cases.

### 4.2. The Chua’s System

**Figure 7:**
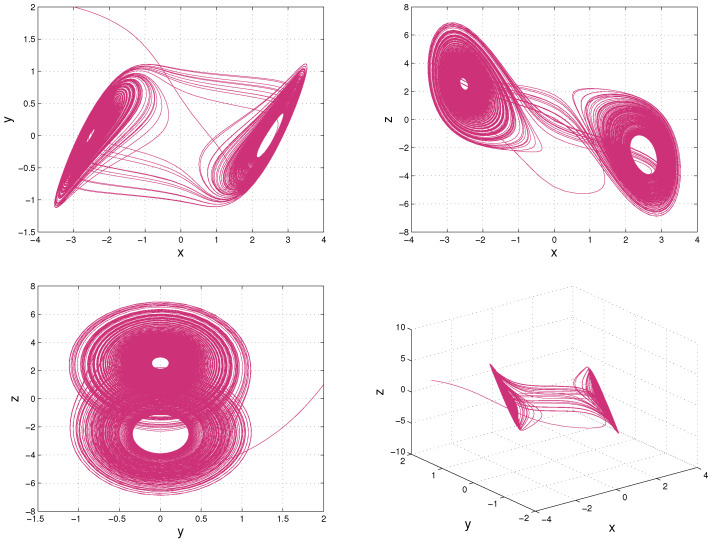
The attractor of Chua’s system with x(0)=−3,y(0)=2,z(0)=1. The former three trajectories are x,z-plane,x,y-plane and y,z-plane, respectively. The last trajectory is a 3D plot of x,y and z.

Since it is the first analog circuit to realize chaos in experiments, the initial Chua’s system is a well-known dynamical model [41]. The Chua’s system is described in reference [42] and there are many researches on its dynamical behavior [43,44]. Here we present an investigation of the information flows within the smooth Chua’s system [45]:

$$\left\{\begin{array}{c} \begin{array}{c} \frac{dx}{dt}=p\left(x+y-x\ln\sqrt{1+x^{2}}\right)\\ \frac{dy}{dt}=x-y+z\\ \frac{dz}{dt}=-qy \end{array} \end{array}\right.,$$

where p,q are parameters, x,y and *z* are state variables in R and $t\in R^{+}.$ When p=11 and q=14.87, a chaotic attractor of the Chua’s system is shown in Figure 7.

As mentioned before, using the same estimation procedures, we can obtain the density ρ(x,y,z) of R by counting the bins at each step. From Figure 7, the appropriate computation domain Ω≡[−10,10]×[−10,10]×[−10,10] which includes an attractor of the Chua’s system can be selected to estimate the three-variable joint probability density function. The following computation is demonstrated by applying a fourth order Runge–Kutta method. Similarly, we only show the results of one experiment after computing information flows multiple times by using different parameters. Suppose that R is distributed as a Gaussian process N(u(t),Σ(t)), with a mean *u* and a covariance matrix Σ in the initial state:

$$u\left(0\right)=\left[\begin{array}{c} 9\\ 9\\ 9 \end{array}\right],\Sigma \left(0\right)=\left[\begin{array}{ccc} 9 & 0 & 0\\ 0 & 9 & 0\\ 0 & 0 & 9 \end{array}\right].$$

**Figure 8:**
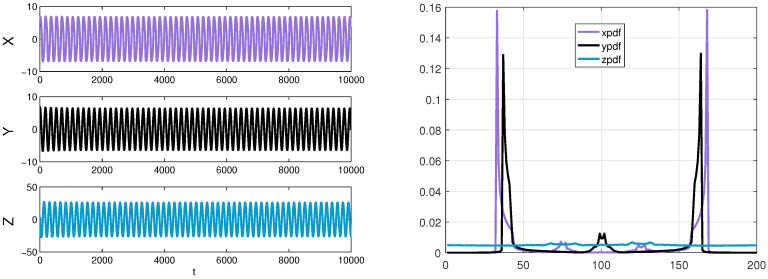
Left panel: a sample data (X,Y and *Z*) of the Chua’s system generated by a fourth order Runge–Kutta method with Δt=0.01; Right panel: the purple line, black line, and blue line represent an estimated marginal density of x,y,z by counting bins, respectively.

**Figure 9:**
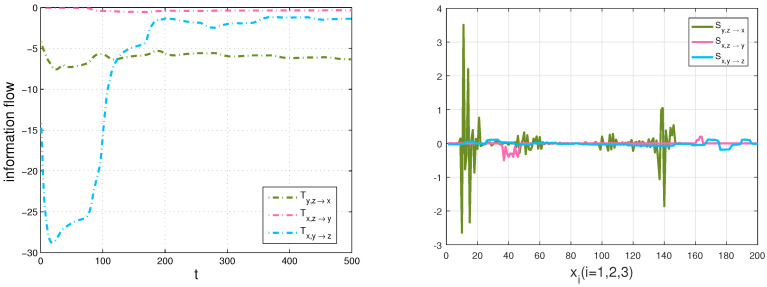
Left panel: the multivariate information flow of the Chua’s system: green dot-dash line: $T_{y,z\to x}$; red dot-dash line: $T_{x,z\to y}$; blue dot-dash line: $T_{x,y\to z}$ (in nats per unit time); Right panel: the information strength of transfer in the Chua’s system: green dot-dash line: $S_{y,z\to x}$; red dot-dash line: $S_{x,z\to y}$; blue dot-dash line: $S_{x,y\to z}$ (arbitrary unit).

Due to the additional fact that the smooth Chua’s circuit has a highly non-coherent dynamics [46], we discretize the sample space into 200×200×200=8,000,000 bins to adequately understand the information transfer and the behavior of the system over time. A sample data and an estimation result of three marginal densities are shown in Figure 8, and we can find that the dynamical behaviors of the system are consistent with the results, such as symmetry. Using formula (12) to compute the information transfers within three variables of Chua’s system. Firstly, we discuss the coupling effect from two components to the other component, the calculation results are demonstrated in Figure 9.

**Figure 10:**
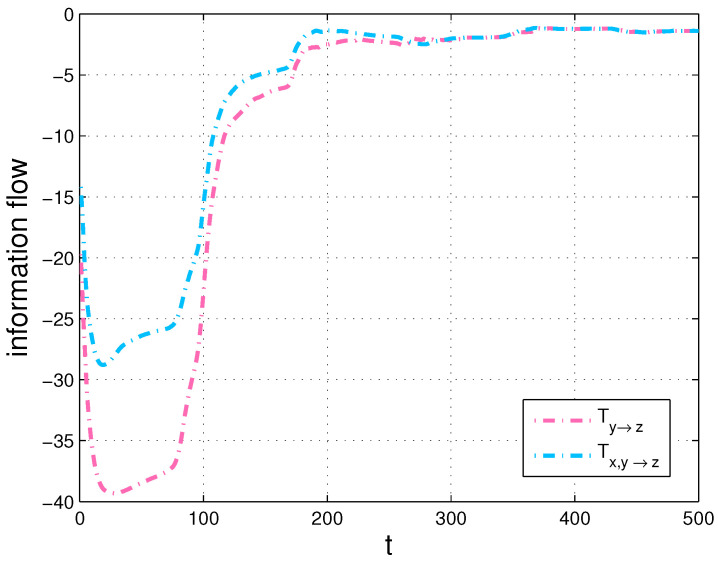
$T_{y\to z}$ and $T_{x,y\to z}$ in the Chua’s system (in nats per unit time).

**Figure 11:**
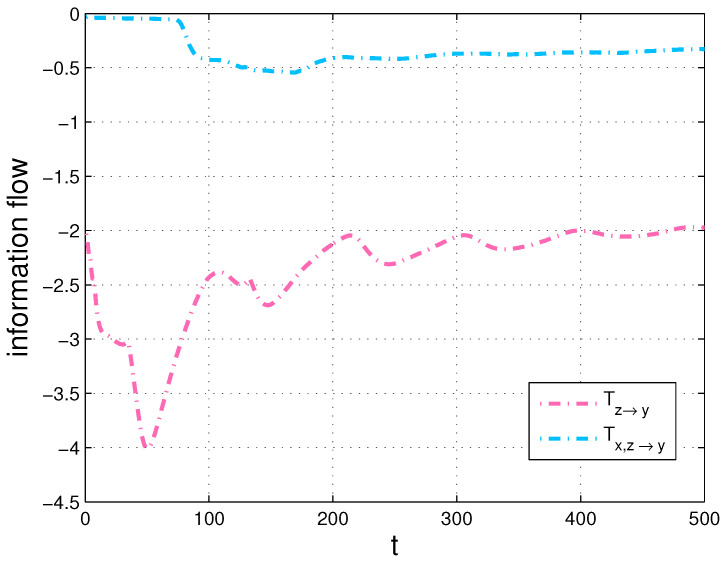
$T_{z\to y}$ and $T_{x,z\to y}$ in the Chua’s system (in nats per unit time).

Secondly, we compute the transfers, $T_{y\to z}$ and $T_{z\to y}$, then compare $T_{y\to z}$ with the transfer, $T_{x,y\to z}$ and $T_{z\to y}$ with $T_{x,z\to y}$ in Figure 10 and Figure 11, respectively. We also show the corresponding results of the strength of information transfer among the components with a Gauss distribution initialization and the averaged density over time in Figure 9.

Since *X* causes *Y* but does not cause *Z* in the Chua’s system, the numerical results of Figure 10 and Figure 11 conform with the derivations of Equations (13) and (14) in Section 3.1. More specifically, there is almost no difference between the two flows in Figure 10, however, there exists large disparity between the two flows in Figure 11. The results also verify our formalisms. In addition, as shown in Figure 10 and Figure 11, we can see that the information flow $T_{y\to z}$ is different from $T_{z\to y}$ due to the asymmetry of information transfer. All simulations are performed on a 64-bit Matlab R2016a environment. We are able to estimate that one variable makes another variable more uncertain or more predictable via the generalized formalisms. Besides, we can identify sensitive variables by computing information transfers among the variables in dynamical systems.

Compared with the Lorenz system, the Chua’s system embodies in engineering systems besides that their discoveries were extraordinary and changed scientific thinking [46]. It can be used as another means to research, experiment and think about humanity, identity and art, etc. [47,48]. In studying visualization of the dynamics of Chua’s circuit through computational models, the quantitative transformations of behavior are being taken into account [46]. The multi-dimensional formalisms of information flow enable us to improve our ability to estimate, predict, and control complex systems in many diverse fields. Furthermore, most existing approaches in control and synchronization of chaotic systems require adjusting the parameters of the model and estimating system parameters, which become an active area of research [49], and an additional benefit provided by the multi-dimensional formalisms of information flow is parameter estimation. We can compute information flows of the simulation model with different sets of parameters and do the same procedure for obtaining a group of feedback, then determine the optimal parameters that cater for the actual needs in order to put insight into complex behavior of models by comparing the change rates.

## 5. Conclusions

Based on the LK2005 formalism, we propose a rigorous and general formalism of the information transfer among multi-dimensional complex dynamical system components, for continuous flows and discrete mappings, respectively. Information transfers are quantified through entropy transfers from some components to another component, enabling us to better understand the physical mechanism underlying the superficial behavior and explore deeply hidden information in the evolution of multi-dimensional dynamical systems. When the generalized formalisms are reduced to 2D cases, the results are consistent with the LK2005 formalism. We mainly focus on the study of 3D systems and apply the formalisms to investigate information transfers for the Lorenz system and the Chua’s system. In the above-mentioned two cases, we show that information flows of the whole evolution and the strength of information transfer at different planes, which implies that how uncertainty propagates and how dynamic essential information in the system transports. The results of experiments on the generalized formalisms conform with observations and empirical analysis in the literature, whose application may benefit many diverse fields. Compared with the qualification of information transfer between two variables [4] and the transfer mutual information method [32], the generalized formalisms are helpful for analyzing the relationships among the variables in dynamical systems and the research of complex systems. Moreover, since the formalism is built on the statistical nature of information, it has the potential to perform sensitivity analysis in multi-dimensional complex dynamical systems and advance our ability to estimate, predict and control these systems. In practice, for complex high-dimensional dynamical systems, it is not easy to give the dynamics analytically. Considering many critical data-driven problems are primed to take advantage of progress in the data-driven discovery of dynamics [35], we are developing a dynamic-free formulation to analyze information flows of multi-dimensional dynamical systems.

In the future, the formalism will be further generated to high-dimensional stochastic dynamical systems and time-delay systems. Meanwhile, future research should investigate how the information flow as a new indicator can be deployed in the frame of dynamic sensitivity analysis.

## Appendix A. Discrete Mappings

Now consider a 3D transformation

(A1) $$\Phi :\Omega \to \Omega ,\left(x_{1},x_{2},x_{3}\right)\to \left(\Phi_{1}\left(x\right),\Phi_{2}\left(x\right),\Phi_{3}\left(x\right)\right)$$

and the Frobenius–Perron operator (F−P operator) $P:L^{1}\left(\Omega \right)\to L^{1}\left(\Omega \right)$ [18] which steers the evolution of its density. Loosely, given a density $\rho =(x_{1},x_{2},x_{3}),$*P* is defined as that

$${\;\int \int \int \;}_{w}P\rho \left(x_{1},x_{2},x_{3}\right)dx_{1}dx_{2}dx_{3}={\;\int \int \int \;}_{\Phi^{-1}\left(w\right)}\rho \left(x_{1},x_{2},x_{3}\right)dx_{1}dx_{2}dx_{3},$$

where *w* represents any subset of Ω. When Φ is invertible, *P* can be expressed clearly as $P\rho \left(\underline{x}\right)=\rho \left[\Phi^{-1}\left(\underline{x}\right)\right]\left|J^{-1}\right|$, where $J^{-1}=J^{-1}\left(x_{1},x_{2},x_{3}\right)=\det\left[\frac{\partial \left(\Phi^{-1}\left(x_{1},x_{2},x_{3}\right)\right)}{\partial (x_{1},x_{2},x_{3})}\right]$ is the determinant of the Jacobian matrix of Φ. Similar to the two-dimensional case, the entropy increases

$$\begin{array}{cc} \Delta H= & -\;\int \int \int \;P\rho \log P\rho dx_{1}dx_{2}dx_{3}+\;\int \int \int \;\rho \log\rho dx_{1}dx_{2}dx_{3}\\ = & -\;\int \int \int \;\rho \left(\Phi^{-1}\left(x_{1},x_{2},x_{3}\right)\right)\left|J^{-1}\right|\log\left[\rho \left(\Phi^{-1}\left(x_{1},x_{2},x_{3}\right)\right)\left|J^{-1}\right|\right]dx_{1}dx_{2}dx_{3}\\ & +\;\int \int \int \;\rho \log\rho dx_{1}dx_{2}dx_{3}\\ = & -\;\int \int \int \;\rho \left(v_{1},v_{2},v_{3}\right)\left|J^{-1}\right|\left[\log\rho \left(v_{1},v_{2},v_{3}\right)+\log\left|J^{-1}\right|\right]\left|J\right|dv_{1}dv_{2}dv_{3}\\ & +\;\int \int \int \;\rho \log\rho dx_{1}dx_{2}dx_{3}\\ = & -\;\int \int \int \;\rho \left(x_{1},x_{2},x_{3}\right)\log\left|J^{-1}\right|dx_{1}dx_{2}dx_{3}, \end{array}$$

concisely rewritten as

(A2) ΔH=Elog|J|.

Meantime, in the case when $\Phi_{k}$ is invertible of 3D transformations,

(A3) $$\Delta H_{k}^{*}=E\log\left|J_{k}\right|.$$

The entropy of $X_{k}$ increases as

(A4) $$\begin{array}{cc} \Delta H_{k}= & -\int_{\Omega_{k}}\left({\;\int \int \;}_{\Omega_{i}\times \Omega_{j}}P\rho dx_{i}dx_{j}\right)\log\left({\;\int \int \;}_{\Omega_{i}\times \Omega_{j}}P\rho dx_{i}dx_{j}\right)dx_{k}\\ & +\int_{\Omega_{k}}\rho_{k}\log\rho_{k}dx_{k}, \end{array}$$

where $\rho_{k}$ is the marginal density of $X_{k}.$

When $\Phi_{k}$ is noninvertible,

(A5) $$\begin{array}{cc} \Delta H_{k}^{*} & =\int \rho_{k}\left(x_{k}\right)\log\rho_{k}\left(x_{k}\right)dx_{k}\\ & -\;\int \int \int \;P_{k}\rho_{k}\left(\Phi_{k}\left(x_{i},x_{j},x_{k}\right)\right)\log P_{k}\rho_{k}\left(\Phi_{k}\left(x_{i},x_{j},x_{k}\right)\right)\rho \left(x_{i},x_{j}|x_{k}\right)\left|J_{k}\right|dx_{i}dx_{j}dx_{k}, \end{array}$$

where $P_{k}$ is the F−P operator when $x_{i},x_{j}$ is frozen as parameters in $P_{k}$. It is easy to find that Equation (A5) reduces to Equation (A3) when $\Phi_{k}$ is invertible. Therefore, the entropy transfers from $X_{i},X_{j}$ to $X_{k}$ can be unified into a form

(A6) $$\begin{array}{cc} T_{i,j\to k}= & -\int_{\Omega_{k}}\left({\;\int \int \;}_{\Omega_{i}\times \Omega_{j}}P\rho dx_{i}dx_{j}\right)\log\left({\;\int \int \;}_{\Omega_{i}\times \Omega_{i}}P\rho dx_{i}dx_{j}\right)dx_{k}\\ & +\;\int \int \int \;P_{k}\rho_{k}\left(\Phi_{k}\left(x_{i},x_{j},x_{k}\right)\right)\log P_{k}\rho_{k}\left(\Phi_{k}\left(x_{i},x_{j},x_{k}\right)\right)\rho \left(x_{i},x_{j}|x_{k}\right)\left|J_{k}\right|dx_{i}dx_{j}dx_{k}, \end{array}$$

where i,j,k=1,2,3 with different i,j,k at the same time.

Just as the former case with continuous variables, the information flow obtained by Equation (16) has the following property:
**Theorem** **A1.** If $\Phi_{k}$ is independent of $x_{i},x_{j}$ in system (A1) with different i,j,k, then $T_{i,j\to k}=0.$

The detailed proof of Theorem A1 is presented in Appendix A.1. Moreover, the formalism of 3D system can be reduced to the formalism in 2D cases with the previously mentioned conditions being satisfied. For example, when $\Phi_{k}$ has no dependence on $x_{i}$,

$$\begin{array}{cc} T_{i,j\to k}= & -\int_{\Omega_{k}}\left({\;\int \int \;}_{\Omega_{i}\times \Omega_{j}}P\rho dx_{i}dx_{j}\right)\log\left({\;\int \int \;}_{\Omega_{i}\times \Omega_{j}}P\rho dx_{i}dx_{j}\right)dx_{k}\\ & +\;\int \int \int \;P_{k}\rho_{k}\left(\Phi_{k}\left(x_{i},x_{j},x_{k}\right)\right)\log P_{k}\rho_{k}\left(\Phi_{k}\left(x_{i},x_{j},x_{k}\right)\right)\rho \left(x_{i},x_{j}|x_{k}\right)\left|J_{k}\right|dx_{i}dx_{j}dx_{k}\\ = & -\int P_{k}\rho_{k}\log P_{k}\rho_{k}dx_{k}\\ & +\;\int \int \;P_{k}\rho_{k}(\Phi_{k}\left(x_{i},x_{j},x_{k}\right)\log P_{k}\rho_{k}\left(\Phi_{i}\left(x_{i},x_{j},x_{k}\right)\right)\left|J_{k}\right|\left(\int_{\Omega_{i}}\rho \left(x_{i},x_{j}|x_{k}\right)dx_{i}\right)dx_{i}dx_{j}\\ = & -\int P_{k}\rho_{k}\log P_{k}\rho_{k}dx_{k}\\ & +\;\int \int \;P_{k}\rho_{k}\left(\Phi_{k}\left(x_{j},x_{k}\right)\right)\log P_{k}\rho_{k}\left(\Phi_{k}\left(x_{j},x_{k}\right)\right)\rho \left(x_{j}|x_{k}\right)\left|J_{k}\right|dx_{j}dx_{k}=T_{j\to k}. \end{array}$$

In particular, when $\Phi_{k}$ has no dependence on $x_{i}$ and $x_{j}$,

$$\begin{array}{c} T_{i,j\to k}=T_{i\to k}=T_{j\to k}=0. \end{array}$$

Furthermore, when $\Phi_{k}$ has dependence on $x_{i}$ and $x_{j}$,

$$\begin{array}{cc} T_{i,j\to k}= & -\int_{\Omega_{k}}\left({\;\int \int \;}_{\Omega_{i}\times \Omega_{j}}P\rho dx_{i}dx_{j}\right)\log\left({\;\int \int \;}_{\Omega_{i}\times \Omega_{j}}P\rho dx_{i}dx_{j}\right)dx_{k}\\ & +\;\int \int \int \;P_{k}\rho_{k}\left(\Phi_{k}\left(x_{i},x_{j},x_{k}\right)\right)\log P_{k}\rho_{k}\left(\Phi_{k}\left(x_{i},x_{j},x_{k}\right)\right)\rho \left(x_{i},x_{j}|x_{k}\right)\left|J_{k}\right|dx_{i}dx_{j}dx_{k}\\ = & -\int_{\Omega_{k}}\left({\;\int \int \;}_{\Omega_{i}\times \Omega_{j}}P\rho dx_{i}dx_{j}\right)\log\left({\;\int \int \;}_{\Omega_{i}\times \Omega_{j}}P\rho dx_{i}dx_{j}\right)dx_{k}\\ & +\;\int \int \int \;P_{k}\rho_{k}\left(\Phi_{k}\left(x_{i},x_{j},x_{k}\right)\right)\log P_{k}\rho_{k}\left(\Phi_{k}\left(x_{i},x_{j},x_{k}\right)\right)\rho \left(x_{j}|x_{k}\right)\rho \left(x_{i}|x_{j},x_{k}\right)\left|J_{k}\right|dx_{i}dx_{j}dx_{k}\\ = & -\int_{\Omega_{k}}\left({\;\int \int \;}_{\Omega_{i}\times \Omega_{j}}P\rho dx_{i}dx_{j}\right)\log\left({\;\int \int \;}_{\Omega_{i}\times \Omega_{j}}P\rho dx_{i}dx_{j}\right)dx_{k}\\ & +\int_{\Omega_{i}}\left({\;\int \int \;}_{\Omega_{j}\times \Omega_{k}}P_{k}\rho_{k}\left(\Phi_{k}\left(x_{i},x_{j},x_{k}\right)\right)\log P_{k}\rho_{k}\left(\Phi_{k}\left(x_{i},x_{j},x_{k}\right)\right)\rho \left(x_{j}|x_{k}\right)\left|J_{k}\right|dx_{j}dx_{k}\right)\\ & \cdot \rho \left(x_{i}|x_{j},x_{k}\right)dx_{i}\\ = & -\int_{\Omega_{i}}T_{j\to k}\cdot \rho \left(x_{i}|x_{j},x_{k}\right)dx_{i}\\ & +\int_{\Omega_{i}}\left(\int_{\Omega_{k}}P_{k}\rho_{k}\log P_{k}\rho_{k}dx_{k}\right)\cdot \rho \left(x_{i}|x_{j},x_{k}\right)dx_{i}-\int_{\Omega_{k}}P_{k}\rho_{k}\log P_{k}\rho_{k}dx_{k} \end{array}$$

The above formalisms can also be generalized to *n*-dimensional systems by efficient processing of the relationship between the F−P operator

$$\int_{w}P\rho \left(x_{1},x_{2},\ldots x_{n}\right)dx_{1}dx_{2}\ldots dx_{n}=\int_{\Phi^{-1}\left(w\right)}\rho \left(x_{1},x_{2},\ldots x_{n}\right)dx_{1}dx_{2}\ldots dx_{n}$$

and the entropy evolution at different time steps. For example, the transfer of entropy from $X_{2},X_{3},\ldots ,X_{n}$ to $X_{1}$ is

$$\begin{array}{cc} T_{2,3,\ldots ,n\to 1}= & -\int_{\Omega_{1}}\left(\int_{\Omega_{2\ldots n}}P\rho dx_{2}dx_{3}\ldots dx_{n}\right)\log\left(\int_{\Omega_{2\ldots n}}P\rho dx_{2}dx_{3}\ldots dx_{n}\right)dx_{1}\\ & +\int_{\Omega }P_{1}\rho_{1}\left(\Phi_{1}\left(x_{1},x_{2},\ldots ,x_{n}\right)\right)\log P_{1}\rho_{1}\left(\Phi_{1}\left(x_{1},x_{2},\ldots ,x_{n}\right)\right)\\ & \cdot \rho \left(x_{2},x_{3},\ldots ,x_{n}|x_{1}\right)\left|J_{1}\right|dx_{1}dx_{2}\ldots dx_{n}. \end{array}$$

Here $\Omega_{2\ldots n}$ is the simplified script of $\Omega_{2}\times \Omega_{3}\times \ldots \times \Omega_{n}.$ Similar to the continuous cases, the generalized version of the property of Theorem A1 is also suitable for multi-dimensional discrete mappings.

### Appendix A.1.

**Proof** **of** **Theorem** **A1** We only need to show that when $\Phi_{k}$ is independent of $x_{i},x_{j}$ in 3D system,

$$\Delta H_{k}^{*}=\Delta H_{k}.$$

According to Equation (A4) and Equation (A5), we only need to prove

$$\begin{array}{c} -{\;\int \int \int \;}_{\Omega }P_{k}\rho_{k}\left(\Phi_{k}\left(x_{k},x_{i},x_{j}\right)\right)\log P_{k}\rho_{k}\left(\Phi_{k}\left(x_{k},x_{i},x_{j}\right)\right)\rho \left(x_{i},x_{j}|x_{k}\right)\left|J_{k}\right|dx_{i}dx_{j}dx_{k}\\ =-\int_{\Omega_{k}}\left({\;\int \int \;}_{\Omega_{i}\times \Omega_{j}}P\rho dx_{i}dx_{j}\right)\log\left({\;\int \int \;}_{\Omega_{i}\times \Omega_{j}}P\rho dx_{i}dx_{j}\right)dx_{k}. \end{array}$$

According to the definition of the F−P operator and the condition that $\Phi_{k}$ is independent of $x_{i},x_{j}$ at the same time,

$$\begin{array}{c} -{\;\int \int \int \;}_{\Omega }P_{k}\rho_{k}\left(\Phi_{k}\left(x_{k},x_{i},x_{j}\right)\right)\log P_{k}\rho_{k}\left(\Phi_{k}\left(x_{k},x_{i},x_{j}\right)\right)\rho \left(x_{i},x_{j}|x_{k}\right)\left|J_{k}\right|dx_{i}dx_{j}dx_{k}\\ =-\int_{\Omega_{k}}P_{k}\rho_{k}\left(\Phi_{k}\left(x_{k},x_{i},x_{j}\right)\right)\log P_{k}\rho_{k}\left(\Phi_{k}\left(x_{k},x_{i},x_{j}\right)\right)\left|J_{k}\right|{\;\int \int \;}_{\Omega_{i}\times \Omega_{j}}\rho \left(x_{i},x_{j}|x_{k}\right)dx_{i}dx_{j}dx_{k} \end{array}$$

because ${\;\int \int \;}_{\Omega_{i}\times \Omega_{j}}\rho \left(x_{i},x_{j}|x_{k}\right)dx_{i}dx_{j}dx_{k}=1$

$$\begin{array}{c} -\int_{\Omega_{k}}P_{k}\rho_{k}\left(\Phi_{k}\left(x_{k},x_{i},x_{j}\right)\right)\log P_{k}\rho_{k}\left(\Phi_{k}\left(x_{k},x_{i},x_{j}\right)\right)\left|J_{k}\right|{\;\int \int \;}_{\Omega_{i}\times \Omega_{j}}\rho \left(x_{i},x_{j}|x_{k}\right)dx_{i}dx_{j}dx_{k}\\ =-\int_{\Omega_{k}}P_{k}\rho_{k}\left(y_{k}\right)\log P_{k}\rho_{k}\left(y_{k}\right)dy_{k}\\ =-\int_{\Omega_{k}}p_{k}\rho_{k}\left(x_{k}\right)\log P_{k}\rho_{k}\left(x_{k}\right)dx_{k}\\ =-\int_{\Omega_{k}}\left({\;\int \int \;}_{\Omega_{i}\times \Omega_{j}}P\rho dx_{i}dx_{j}\right)\log\left({\;\int \int \;}_{\Omega_{i}\times \Omega_{j}}P\rho dx_{i}dx_{j}\right)dx_{k} \end{array}$$

where $y_{1}=\Phi_{k}\left(x_{k},x_{i},x_{j}\right).$ So

$$\begin{array}{cc} \Delta H_{k}^{*}= & \int \rho_{k}\left(x_{k}\right)\log\rho_{k}\left(x_{k}\right)dx_{k}\\ & -\;\int \int \int \;P_{k}\rho_{k}\left(\Phi_{k}\left(x_{k},x_{i},x_{j}\right)\right)\log P_{k}\rho_{k}\left(\Phi_{k}\left(x_{k},x_{i},x_{j}\right)\right)\rho \left(x_{i},x_{j}|x_{k}\right)\left|J_{k}\right|dx_{i}dx_{j}dx_{k}\\ = & -\int_{\Omega_{k}}\left({\;\int \int \;}_{\Omega_{i}\times \Omega_{j}}P\rho dx_{i}dx_{j}\right)\log\left({\;\int \int \;}_{\Omega_{i}\times \Omega_{j}}P\rho dx_{i}dx_{j}\right)dx_{k}\\ & +\int_{\Omega_{k}}\rho_{k}\log\rho_{k}dx_{k}=\Delta H_{k}. \end{array}$$

 □
